# Practical Things with Illustrations

**Published:** 1897-11

**Authors:** J. G. Templeton

**Affiliations:** Pittsburg, Pa.


AMERICAN JOURNAL

—OF—

DENTAL SCIENCE.

VOL. XXXI. BALTIMORE, NOVEMBER, 1897. NO. 7.

ARTICLE I.

### PRACTICAL THINGS WITH ILLUSTRATIONS.

BY PROF. J. G. TEMPLETON, PITTSBURG, PA.

Read before Ontario Dental Society.

In your programme, I see that one of the subjects is "Popular Dental Education." Now there are so many ways to educate people, and we obtain our education through so many different channels, that we have thought to suggest two things as being worthy of particular attention by our profession at the present time. They are, first, a much better preliminary education as a qualification for entering our profession; and, second, "The *suppression of the horrible* in dentistry."

However, I suppose that the meaning of the phrase, "popular dental education," has reference to the laity, so to speak, or the people at large. As it is well known to us that there is scarcely anything with which they have more to do, and yet about which they possess so little correct knowledge. Now in reference to the best method of disseminating the proper information, it has long been in the mind of the writer that the best method for the diffusion of correct ideas concerning the duties and operations of the dentist, would be carefully prepared lectures delivered before all our teachers' associations, supplemented by giving the same before the advanced classes in all our schools.

*The Making of Artificial Dentures a Fine Art.*

Yes, so it is. But when we go among the people and see the horrible imitations called artificial teeth in use by the people, we are inclined to think that the fine art part of what we see is quite a joke. Hence the conclusion, that the suppression of the horrible is greatly needed. It is no honor to us, that in this age of progress in the arts and sciences, so many evidences of the low standard of art in prosthetic dentistry should be seen everywhere we go. Yes, *false teeth* everywhere; they grin at us in the street, at church, in the theatre, everywhere, like a horrible nightmare, while the poor, unsuspecting victim seems to enjoy their hideousness. No wonder artificial teeth can be had for such a low compensation; they all look alike—like a job-lot, for this reason educated people have a perfect horror of them and feel robbed if they have to pay anything for them. One great defect of many sets of teeth, as we see them in use, is their youthful appearance; they look as if they had been borrowed from some younger person. The dental artist, like the sculptor and portrait painter, has the artist's license to make his subject look a little younger and, if possible, more beautiful; but if he should err by carrying this too far, he then produces a caricature and the result is just the opposite of what he hoped to accomplish, and from what we often see, we are very often inclined to think that many dentists must be color blind or else we would not see so many sets of artificial teeth the color of well watered skim-milk. The remedy is, educate the people æsthetically.

*To Keep Instruments Nicely Polished.*

Burnishers give better results when new than when tarnished, and it is essential to keep them finely polished; in fact, it is desirable to keep all instruments polished. An efficient device for polishing can be made by fastening a piece of sole leather on a block of wood of suitable size and placing a little diamantine powder on this surface. Diamantine is used by jewelers and can be obtained from them or from their supply houses. Diamantine is nothing more or less than oxide of tin, and can be obtained from a wholesale drug house for about sixty cents a pound.

*To Make Gutta Percha Fillings Moisture Tight.*

Dry the cavity well, place in it a pellet of cotton saturated with absolute alcohol; remove the cotton, and with a warm air syringe, evaporate the alcohol; varnish the cavity with a solution of common resin in chloroform, warm the gutta-percha and pack into the cavity with a cold instrument; heat a thin bladed instrument and pare off the surplus filling, after which a fine polish can be given to it by rubbing with a little oil of cajeput.

*Placing Rubber Dam on Lower Front Teeth.*

A slip noose can be put on the lower front teeth with one hand, while the rubber dam is held down with the other; get the slip knots ready first, draw them tight and they will hold as long as wanted.

*The Use of Beads.*

The use of clamps can very often be avoided in filling teeth by tying one or two small beads near the middle of the string used as a ligature; after placing the rubber dam, tie the ligature so that the beads will come on the lingual side of the tooth and the rubber dam will not slip off over the beads.

*Excavators.*

The writer is of the opinion that the old fashioned excavator and bur drill should be used in the preparation of cavities much more than they are now; we are inclined to think that the engine is relied on too much for this purpose.

### *Thermal Changes.*

To protect from thermal changes, particularly in deep cavities where the pulp is not quite exposed, first dry with bibulous paper, then apply, on a small pellet of cotton, absolute alcohol which has a strong affinity for any moisture that may be left in the cavity or open ends of the tubuli; when the cotton is removed, evaporation takes place rapidly, leaving the cavity perfectly dry. Now varnish inside of cavity to near the margin with a solution of common resin in chloroform or of gum sandarach, dissolved in sulphuric ether, then take a small piece of asbestos felt, moisten with pure wood creosote camphophenique, or oil of Eucalyptus, and cover the side to go next to the pulp with a mixture of iodol oxide of zinc and vaseline; after this is in position in the cavity, place over it a thin piece of lead or a thin piece of aluminum plate, which will prevent pressure against the most vulnerable point in the bottom of the cavity, while inserting either a gold or other metallic filling. We often cover the bottom of deep cavities with a jelly made by mixing carbolic acid and colodian together, then absorb the acid with cotton or bibulous paper, which leaves the colodian residuum of a leather-like substance, and over which we place the asbestos, and we think we have an excellent remedy against thermal changes.

### *To Take a Perfect Impression for Partial Upper Plate.*

To take an accurate impression of the mouth for a partial upper set of teeth, smear plaster over the roof of the mouth with the finger, take a string about a foot in length; tie the ends together, put the tied ends of the loop into the plaster on the roof of the mouth and add more plaster to thoroughly imbed the knot, leaving loop of string hanging down. In placing the plaster in the mouth, care should be taken to have it come full half way over the grinding surfaces of molars and bicuspids and also cutting edges of the front teeth; then trim the plaster and varnish the trimmed surfaces. The plaster should be so trimmed that it will fill up fully one half of all spaces between the teeth; then cover all the remaining surface of the mouth and teeth with plaster, being very careful to have the teeth well covered and spaces filled in, putting plaster for the buccal and labial surfaces. When set, the plaster impression readily parts where it has been varnished, the palatial portion is dislodged with the help of the string used, and the pieces are then placed together and model made. If a tooth is irregular, use modeling compound about it and trim suitably, then apply the plaster. When removing, it breaks where joined; then remove compound, place in position in the impression and pour the model. Before pouring, the impression should be coated with a lather of soap and then immersed in water for about ten or fifteen minutes. When the plaster has had sufficient time to set, separation can be made, and a model thus obtained will not have any of the fine lines obliterated.

### *Articulating Teeth.*

Always take an impression of the lower teeth when making an upper set, and in taking the bite, have wax trimmed to show the length you wish the teeth to be, and bite into it just sufficiently to show the tips of cutting edges and cusps where the model made from lower impression can be placed in proper position, etc. For double sets, make wax models for contour in restoration of features and to show length of teeth, and then try these models in the mouth, being careful to see that you have it right; then make plaster articulating models for setting up the teeth, setting up the lower ones first against a plaster articulating plate, its articulating surface corresponding with the articulating surface of lower wax model; then lay aside the plaster articulating plate and put the model of upper jaw in its place and set the upper teeth to the lower ones. The writer adopted this method about twenty-eight years ago, and in that length of time has not had to grind a cusp off to let the front teeth come together. But a more elaborate description of this seems to be required in order that it may be understood and adopted as a practical method. Having to make a full upper set of teeth, we will suppose the impression and model to have been made in the usual way. Take modeling composition, soften and flatten out until it is about a quarter of an inch thick, press it on the model while warm and then cut and trim to make a trial plate for the purpose of taking a bite. It should fit the model accurately. Melt a little wax around on the ridge, then press a roll of softened wax on that and trim to what you think would be a sufficient length, then try in the mouth and carefully trim the lower edge to the proper length for the teeth; if it is not, either add to or cut away until it is found by trying in the mouth that the wax represents the proper length. This wax should be so cut on its articulating surface that all the lower natural teeth will strike at the same time when tried in the mouth. Now remove and soften the articulating wax surface just a little over the flame, then replace in the mouth and do not let the patient bite into it until you have the head drawn well back so as to put the anterior muscles of the neck on a stretch; then have the patient bite a little on the wax just to get an impression of the cusps and cutting edges of all the lower teeth. Next take an accurate impression of the lower teeth, from which make a plaster model which will fit into the slight impressions of the teeth made in the bite taken, and then place the whole on any good articulator which can be set to maintain the relative positions. Remove the bite and you are ready to set the teeth to a correct articulation, and if all has been carefully done, the teeth will come together properly without any subsequent grinding.

For a double set (upper and lower) make trial plates of modeling composition to take the bite on, putting a piece of rather stiff wire in the lower one to stiffen it. Wax the ridges as previously described. Place a roll of softened wax on the upper trial plate, place the lower trial plate in the mouth, being careful to see that it is in its proper place, and hold it there while putting in the upper plate with the wax on it. Do not allow the patient to bite until the head is thrown back as far as you can get it, then tell the patient to bite, and keep the jaws closed until with the finger the wax has been well pressed on to the trial plates. Make the centre or medium line on the wax. Have patient close the lips, and then take a small straight instrument and mark on the wax the height of the lower lip. This mark should extend from one angle of the mouth to the other; you then have the line of fissure or line of lip closure; in other words, the height of the lower lip and length of the upper to serve as a guide in making the wax models. After thus taking the bite, place each of the models in the bite so obtained, and fasten in any good articulator; then prepare corresponding wax models, which should be tried in the mouth to verify their correctness. They should come together in the mouth the same as on the articulator, and if they do not they should be made to do so before proceeding further. Take particular pains to be satisfied that the wax models are correctly adjusted and give a natural expression to all the facial features, observing that the lower third of the face is in proper proportion or length with the upper two-thirds, and be sure to produce the proper fullness over the region of the upper cuspids to give as near as possible the natural contour. Then take the upper and lower plaster models off the metal articulation, and make a plaster extension to the back part of the upper model, on which place the wax models, which have been marked while in the mouth, so that they can be put in the same position out of the mouth. The lower plaster model is placed in position, and a plaster extension added to fit to that of the upper plaster model. After separating these, the lower wax model is placed on the lower plaster model, and the inside space filled with wet paper, and plaster is poured over all to make the lower articulating plate to which the lower teeth are to be set. Next place the upper model in position, after the lower teeth have been set, and set the upper teeth to the lower ones which have just been set to the lower articulating plate. Always set the lower teeth first. And in setting the upper teeth always set the bicuspids first.

Having made double sets in this way for twenty-eight years without having to do any grinding after placing them in the mouth, the writer is inclined to think that he has some claim to the conclusion that this method is a pretty good one.

To take a plaster impression with a small quantity of plaster, first take an impression in wax, and then cut away the palatal, buccal and labial surfaces to almost an eighth of an inch in depth, and over the surfaces thus formed out slight lines in different directions, with about two teaspoonfuls of plaster spread evenly over said surfaces, and reinserted and held in place until the plaster has well set, and you have obtained an impression that has no thick body of plaster in any one place to cause uneven shrinkage.

*To Duplicate Models and Impressions.*

Take printer's ink roller composition, heat in a water bath until well melted. Grease the model slightly with lard, and place it the same as if to mould a metal die, cover with a metal ring (a tin can opened at both ends will do), and pour the melted composition over the model. Let this stand over night. By morning the material is hardened, and the model can be withdrawn. The composition being elastic it retains its shape, and a hundred models may be poured if necessary.

To prevent plaster from adhering to the palatine surface of vulcanite plate. Just before packing the case coat the model with a thick solution of soap, almost any kind of soap will do, but that which makes a thick lather in the shortest time is the best.

*To Line Vulcanite Plates with Black Rubber.*

Before packing, coat the cast three or four times with a solution of black rubber, allowing each to harden or dry before applying the next. In swaging aluminum plates, always keep the plates between two pieces of silk tissue paper, to prevent its coming in contact with either the lead or zinc, either of which metals is injurious to it. In swaging any kind of metal plate the writer has been in the habit of first stamping that portion of the plate which is to fit to the roof of the mouth, and the rest afterwards, as we shall show in our illustration.

*Models of Marble Dust and Plaster.*

In vulcanite work the best results may be obtained by making models one-fourth marble dust and three-fourths plaster; also the same in flasking the case.

*To Prevent Rubber Runniug Between Joints.*

To keep rubber from running between the teeth and joints in vulcanizing, after the teeth are set in the first half of the flask, plaster, trimmed and varnished, pour water on all the teeth and joints, then mix a small quantity of pure plaster, have it rather thin, and with mixing spatula cover labial and buccal surfaces, also the joints, take up the piece quickly and bring it near the mouth, and blow rather sharply against the thin plaster all around, which will force it into all spaces between the teeth or blocks. After this finish flasking in the usual way, and, if possible, it is well to allow the case to remain over night in the flask before packing.

*To Trim Rubber Plate.*

In finishing vulcanite plates always trim the rim low over the bicuspids, leaving it high as can be worn over the cuspids, and the same over and back of the second molars; do not file rim to a knife-like edge, slightly bevel inside of rim at the top, extending down about three-sixteenths of an inch. In upper plates the back part, or that portion over the second molar should be left as high as it can be worn, and in many cases the rim should project over the second molars; and also over the cuspids, such projections are often a very material help in retaining plates, particularly in that class of cases generally considered as unfavorable for retention.

### TO MAKE PLATINUM AND GOLD PLATE.

To make platinum and gold plate, melt with blow pipe on a piece of platinum plate pure gold, and roll to desired thickness; the result will be as good as any you can buy.

### GOLD SOLDERS.

Take a United States $5 gold piece, 20 grains coin silver, 10 grains pure copper, 6 grains English toilet pins; melt the silver and copper together first; after melting this and the gold together, add the pins, flow into an ingot and roll, cut into small pieces and melt again if it does not roll well first time; this will give a solder a little more than 19 carats fine, and flows nicely on coin gold, being the same color. This we call No. 1. Now take of

| | |
|---|---|
| No. 1, | 89 grs. |
| Coin silver, | 7 " |
| Pure copper, | 4 " |

Melt together and roll, and we have a second grade which we call No. 2, and which will flow on No. 1. To make a still lower grade take:

| | |
|---|---|
| Pure gold, | 6 dwt. |
| Copper, | 2 " |
| Fine silver, | 1 " |

And you will have a 16 carat solder. In my practice only Nos. 1 and 2 are used, and are made according to the form 1 as given above.

### TO SOLDER A CAP ON GOLD CROWN.

To solder a cap on a gold tube intended for an artificial crown lay the cap on about a tablespoonful of finely cut asbestos, put the tube in place on the cap, drop in the solder and a little powdered borax, then blow a yellow flame on the asbestos, all around the tube until the solder flows, and there will be no danger of melting the gold.

### TO HARDEN PLASTER BOIL IN PARAFFINE.

To give your plaster casts or models the appearance of ivory boil them in pure white wax.—*Dominion Dental Journal.*

---

ARTICLE II.

